# Are There Benefits of Total Hip Arthroplasty with Dual-Mobility Cups Compared to Bipolar Hemiarthroplasty for Femoral Neck Fractures in the Geriatric Population? A Systematic Review and Meta-Analysis of Comparative Studies

**DOI:** 10.3390/jcm14145076

**Published:** 2025-07-17

**Authors:** Dimitrios Grammatikopoulos, Vasileios F. Pegios, Stavros Tsotsolis, Eustathios Kenanidis, Eleftherios Tsiridis

**Affiliations:** Academic Orthopaedic Department, Aristotle University Medical School, General Hospital Papageorgiou, Ring Road Efkarpia, 56403 Thessaloniki, Greece; dimigram@auth.gr (D.G.); vfpegios@auth.gr (V.F.P.); stavros.tsotsolis@nhs.net (S.T.); ekenanidis@auth.gr (E.K.)

**Keywords:** femoral neck fractures, frail elderly, hemiarthroplasty, total hip arthroplasty, survival, complications, reoperation, meta-analysis

## Abstract

**Background/Objectives**: The optimal treatment for femoral neck fractures (FNFs) in the elderly remains unclear. Internal fixation, bipolar hip hemiarthroplasty (BH), standard total hip arthroplasty (THA), or dual mobility (DM-THA) cups have been employed, each presenting various advantages and disadvantages. This systematic review and meta-analysis evaluated comparative studies of BH and DM-THA in FNFs among the elderly, aiming to ascertain differences in outcomes, including functional recovery, patient-reported outcome measures, implant survival, complications, and mortality rates. **Methods**: This meta-analysis followed PRISMA 2020 guidelines with a pre-registered PROSPERO protocol (CRD420251065762). A comprehensive search of electronic databases and grey literature included only comparative studies of BH and DM-THA in patients over 65 years with FNFs. **Results**: Sixteen studies were eligible, comprising four randomised controlled trials and twelve retrospective comparative studies involving 11,460 patients (10,036 BH; 1424 DM-THA). Patients with DM-THA exhibited a higher postoperative Harris Hip Score (4.55, *p* < 0.0001), alongside a lower dislocation risk ([OR] 2.77, *p* < 0.0001), a reduced revision rate ([OR] 2.36, *p* < 0.0001), and decreased mortality ([OR] 1.94, *p* < 0.0001). The operative time was somewhat longer in the DM-THA group, by 12.71 min, and blood loss was greater by 121 mL, indicating significant heterogeneity across the studies. **Conclusions**: DM-THA for FNFs in elderly patients results in improved functional recovery and lower dislocation, reoperation, and mortality risk. However, longer operative times and increased blood loss remain significant considerations. Further, well-designed comparative studies are required to evaluate overall cost-effectiveness and define the optimal age threshold, beyond which the limitations of DM-THA may outweigh its benefits.

## 1. Introduction

Femoral neck fractures (FNFs) are among the most prevalent fractures in older adults, usually due to low-energy falls from a standing position [1,2]. Their prevalence in Western populations has seen a surge in recent years [3]. FNFs pose a serious threat, particularly considering the already compromised health of elderly patients, potentially leading to multiple complications and a high mortality risk, which may be as high as 20% within the first year following the injury [1,2].

Elderly patients with displaced FNFs, especially those over 75, have often undergone hip hemiarthroplasty (HA) [4]. This procedure involves substituting the fractured femoral head with a prosthetic stem and head, keeping the native acetabulum intact. For decades, bipolar heads have been recognised as the gold standard in hip HA, offering improved range of motion, greater stability, and lower dislocation risk compared to earlier unipolar models [5,6,7]. Total hip arthroplasty (THA) has typically been reserved for patients aged 65 to 75, although the primary factors influencing the choice of surgery are patient comorbidities and overall health [8,9,10].

Dual mobility (DM) cups are a well-established concept in THA and resemble the bipolar hemiarthroplasty (BH). These cups contain a large polyethylene bearing that encases a smaller femoral head, like the BH, which moves within a metal shell in the acetabular cup, allowing an additional degree of movement [11,12,13]. While DM cups are utilised in both primary and revision THA, they are often chosen to address hip instability, particularly for patients with severe hip dysplasia, spinopelvic imbalances, neuromuscular disorders, or cognitive disabilities [11,14].

Considering these advantages, there has been growing interest in using Dual Mobility Total Hip Arthroplasty (DM-THA) to manage displaced FNFs as a viable alternative to the established BH. This interest is driven by a demographic shift towards a more active elderly population that necessitates enhanced functional recovery and more durable implants. The principal advantage of DM cups in this population is their heightened hip stability, especially in light of recent research demonstrating an increase in revision rates for BH [15,16]. Furthermore, reducing the frequency of revision surgeries can significantly alleviate the long-term socioeconomic burden of these injuries [17,18]. Direct comparisons between these two procedures in the past have highlighted these potential benefits of DM-THA [19]. Conversely, prevailing concerns regarding increased operative time, blood loss, length of hospital stay, and upfront costs remain for this population [20]. The optimal surgical approach for the geriatric population with FNFs remains uncertain, as does the age threshold beyond which the implementation of DM cups fails to yield additional benefits.

This systematic review and meta-analysis aimed to ascertain differences in outcomes, including functional recovery, patient-reported outcome measures (PROMs), implant survival, complication and mortality rates, and the need for revision surgeries between bipolar BH and DM-THA for displaced FNFs in elderly patients, focusing solely on comparative studies of these two treatment types.

## 2. Materials and Methods

### 2.1. Literature Search Strategy 

This systematic review was conducted according to the Preferred Reporting Items for Systematic Reviews and Meta-Analyses (PRISMA 2020) [21] statement and in line with the protocol agreed to by all authors. A PRISMA checklist is included in Supplementary Materials S1. The review protocol was registered in the International Prospective Register of Systematic Reviews (PROSPERO) under registration number CRD420251065762. Studies were identified by searching the PubMed database, ScienceDirect, and the Cochrane Library (end of search date: June 2025) by two reviewers (DG, ST). The following search terms, along with the appropriate MeSH terms, while employing Boolean operators, were used: -(((((dual mobility [Title/Abstract]) OR (DM[Title/Abstract])) OR (Tripolar [Title/Abstract])) OR (acetabular component [Title/Abstract])) OR (cup [Title/Abstract])) OR (“Arthroplasty, Replacement, Hip”[Mesh])-((((“Hemiarthroplasty”[Mesh]) OR (bipolar [Title/Abstract])) OR (hemiarthroplasty*[Title/Abstract])) OR (partial hip [Title/Abstract])) OR (hemi [Title/Abstract])-((((((((“Hip Fractures”[Mesh])) OR (intracapsular fracture*[Title])) OR (hip fracture*[Title])) OR (neck fracture*[Title])) OR (non-elective [Title])) OR (femoral [Title])) OR (femur [Title]))-(((((((comparative [Title/Abstract]) OR (comparison [Title/Abstract])) OR (outcome*[Title/Abstract])) OR (risk [Title/Abstract])) OR (survival [Title/Abstract])) OR (assessment [Title/Abstract])) OR (“Treatment Outcome”[Mesh])) OR (“Treatment Expectations”).

The exact search string that was used is presented in Supplementary Materials S2. The reference lists of relevant published articles were manually searched to identify any missing records. All identified citations were imported into Zotero [22] for reference management. After removing duplicates, three reviewers (DG, ST, VP) independently screened the titles and abstracts to identify potentially eligible studies. Studies that appeared to meet the inclusion criteria or those for which eligibility could not be determined from the abstract were retrieved in full text. Discrepancies during the selection process were resolved through discussion, and if consensus was not achieved, a fourth senior reviewer (EK) was consulted.

### 2.2. Inclusion and Exclusion Criteria 

Eligible studies were required to be randomised controlled trials (RCT) or comparative observational studies, both prospective and retrospective, involving adult patients aged 18 years or older with displaced intracapsular FNFs. The intervention of interest was BH, while the comparator was DM-THA. Studies needed to report at least one relevant comparative clinical outcome, including dislocation rate, reoperation rate, mortality rate, or functional scores such as the Harris Hip Score. We excluded studies that involved only monopolar HA or conventional THA without DM components, as well as case reports, editorials, animal or cadaveric studies, and articles that lacked full-text availability or were not published in English.

### 2.3. Data Extraction

Two reviewers independently extracted data from each included study using a standardised data collection form. The extracted information encompassed study design, year of publication, sample size, patient demographics (age and sex), type of surgical intervention (BH or DM-THA), fixation method, duration of follow-up, and reported clinical outcomes. These outcomes included blood loss, dislocation rates, revision surgeries, mortality, and functional scores. Any discrepancies in data extraction were resolved through full-text discussions among all reviewers. No unresolved disagreements arose during this process.

### 2.4. Risk of Bias and Study Quality Assessment

Two reviewers (DG and ST) independently evaluated the methodological quality and risk of bias of each included study using the Cochrane Risk of Bias 2 assessment tool (RoB2) for randomised controlled trials [23] and the Newcastle–Ottawa Scale (NOS) for retrospective cohort studies [24]. The evaluation focuses on selection bias, comparability, and assessment of outcome measures. The results of the quality assessment are exhibited in Supplementary Materials S3 and S4, respectively. Any disagreements were resolved by consulting a senior reviewer (EK).

### 2.5. Statistical Analysis

Six meta-analyses were performed, one for each of the following comparative outcomes between BH and DM-THA patients: (i) operative time, (ii) estimated blood loss, (iii) postoperative Harris Hip Score, (iv) dislocation rate, (v) reoperation rate, and (vi) mortality rate. For the outcomes based on continuous variables (i, ii, iii), a random effects model with inverse-variance weighting for estimation of mean difference was implemented. The confidence interval (CI) was set at 95%. Five studies did not provide data for the standard deviation. Based on the nature of the studied variables and on data from the remaining studies, it was assumed that these data followed a normal distribution. Consequently, a standard deviation was imputed based on the mean value of the standard deviations of the remaining included studies. For the outcomes that contained dichotomous data (iv, v, vi), a random effects model with inverse-variance weighting was employed, and the odds ratio (OR) was used as an effect measure with a 95% confidence interval. The I2 statistic assessed heterogeneity. All data analyses were conducted using Review Manager (RevMan) Version 5.4., Copenhagen, The Cochrane Collaboration, 2020 [25].

## 3. Results

### 3.1. Search Results

**Figure 1:**
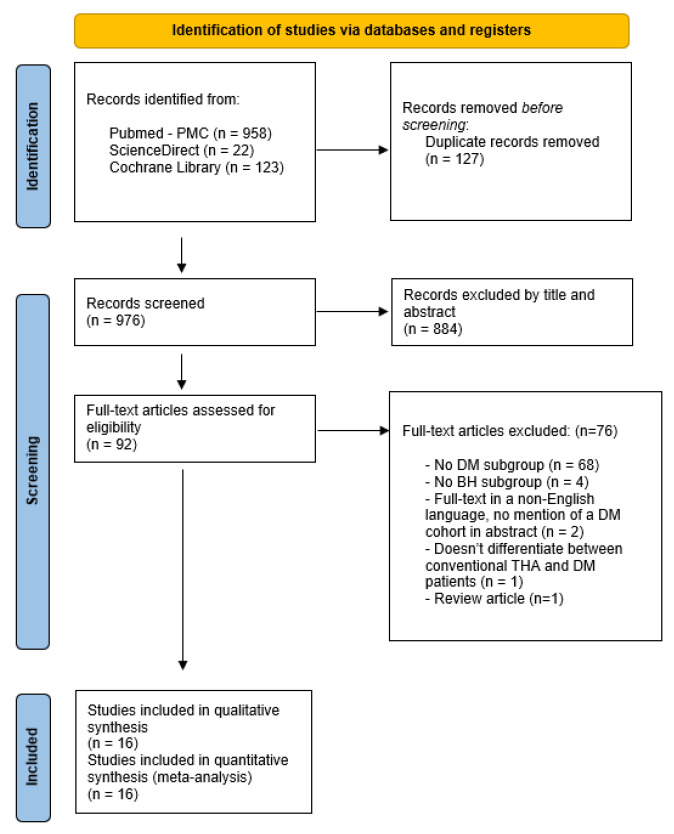
Preferred Reporting Items for Systematic Reviews and Meta-analyses (PRISMA) flowchart. DM: Dual Mobility Total Hip Arthroplasty, BH: Bipolar Hemiarthroplasty, THA: Total Hip Arthroplasty.

The initial search identified a total of 1103 studies. After removing duplicates, 976 studies were screened, and 884 of these were excluded based on their titles and abstracts. The full texts of the remaining 92 studies were evaluated comprehensively. Ultimately, 16 studies [26,27,28,29,30,31,32,33,34,35,36,37,38,39,40,41] were deemed suitable for inclusion in both the qualitative and quantitative syntheses. A PRISMA flow diagram illustrating the study screening and selection process is depicted in Figure 1. The studies that were excluded after full-text evaluation, along with the reasons for their exclusion, are displayed in Supplementary Materials S4.

### 3.2. Included Studies Design and Patient Demographics

The included studies were published between 2014 and 2025. Twelve of these were retrospective comparative studies [26,27,28,29,30,33,35,36,37,38,39,41], while the remaining four were randomised controlled trials [31,32,34,40]. All studies featured at least two subgroups of patients who underwent surgical treatment for an FNF, either with bipolar HA or with THA with a DM cup, and two or more comparative outcomes between these two subgroups were assessed. A total of 11,460 patients across sixteen studies were included in the analysis; 10,036 patients were treated with BH and 1424 patients with DM-THA. The average ages of patients in the BH and DM-THA subgroups were 79.3 and 76.5 years, respectively, at the time of surgery. The percentage of male patients who underwent BH was 32.9%, whereas the percentage of male patients who underwent DM-THA was 30.7% (Table 1).

### 3.3. Studied Outcomes and Follow-Up

Seven studies [26,27,29,32,34,40,41] provided data on the estimated blood loss during the operation for each treatment group. Fifteen, 10, and 13 studies examined dislocation rates [26,27,28,29,31,32,33,34,35,36,37,38,39,40,41], reoperation rates [26,27,28,31,32,33,35,38,39,41], and mortality rates [26,27,28,29,30,31,32,35,36,37,38,40,41] between the groups, respectively. Eight studies measured the postoperative Harris Hip Scores for each group [29,30,32,33,34,39,40,41]. Follow-up varied across studies, ranging from 6 to 62 months. However, 14 studies included a minimum follow-up of 12 months for both groups of patients [26,27,28,29,30,31,32,33,34,35,36,38,40,41] (Table 2).

### 3.4. Operative and Implant Characteristics

All but one study provided information on the surgical approach. A variety of approaches were employed, with the posterolateral approach being the most common. The direct anterior approach, the anterolateral approach, and the direct lateral approach were also utilised in some of the studies [27,30,31,36,39,40]. Eleven studies reported the mean operative time for each type of surgery [26,27,28,29,31,32,34,37,39,40,41]. Furthermore, most studies reported the type of implants used and the type of fixation for the femoral stem in both procedures, the acetabular component for DM-THA, and other technical aspects of the operations. The allocation of patients to each type of surgical treatment varied based on study design and institution-specific protocols. These details are summarised in Table 3.

### 3.5. Cognitive and Neuromuscular Status of Patients in the Included Studies

Nine studies assessed the cognitive and neuromuscular status of the included patients at the time of surgery [28,29,31,32,33,35,36,38,39,41]. Specifically, three studies excluded all patients with either cognitive impairment or a neuromuscular deficit that could affect walking ability [29,39,41]. Conversely, two studies included only patients diagnosed with cognitive impairment or a neuromuscular disorder [33,36]. Finally, the study by Iorio et al. included only patients diagnosed with dementia, but not with a neuromuscular disorder [31]. A diagnosis of cognitive impairment involved Alzheimer’s disease or other dementias, and systemic CNS atrophy. Neuromuscular disorders comprised Parkinson’s disease, myasthenia gravis, other myopathies, epilepsy, and multiple sclerosis (Table 4).

### 3.6. Outcomes

Six meta-analyses were performed, one each for the following comparative outcomes between BH and DM-THA patients:

(i) Operative time

**Figure 2:**
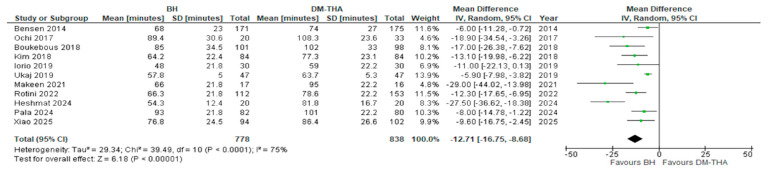
Forest plot for comparison of Operative Time [26,27,28,29,31,32,34,37,39,40,41].

The average length of the operation for each group was reported in 11 studies [26,27,28,29,31,32,34,37,39,40,41]. As expected, the operative time was higher for DM-THA in every study. However, there were disparities regarding the mean difference in surgical time between the two procedures, with two studies displaying an increase of only 6 min [26,27,28,29,30,31,32]. In contrast, in the study by Makeen et al., the duration of surgery increased by 29 min when performing a DM-THA [34]. The overall mean difference was statistically significant (12.71, 95% CI: 8.68 to 16.75, *p* < 0.0001). The *I^2^* index was 75% (Figure 2).

(ii) Estimated blood loss

**Figure 3:**
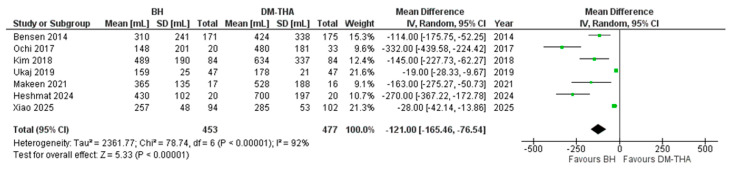
Forest plot for comparison of Estimated Blood Loss [26,27,29,32,34,40,41].

Seven studies recorded data on the average estimated blood loss during surgery [26,27,29,32,34,40,41]. Similarly to operative time, DM-THA averaged a higher volume of intraoperative blood loss compared to BHA, as was anticipated, yet the difference in the estimated amount of blood that was lost between BHA and DM-THA exhibited considerable variability among studies, with studies reporting mean differences as low as 19 mL [32] and as high as 332 mL [27]. The overall mean difference was statistically significant (121.00, 95% CI: 76.54 to 165.46, *p* < 0.0001). The heterogeneity of the studies was considerable (*I^2^* = 92%) and statistically significant (Figure 3).

(iii) Postoperative Harris Hip Score (HHS)

**Figure 4:**
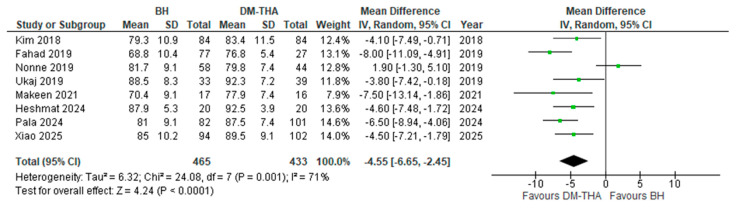
Forest plot for comparison of Postoperative Harris Hip Score [29,30,32,33,34,39,40,41].

Eight studies utilised the HHS to evaluate the functional status of the patients at various postoperative intervals [29,30,32,33,34,39,40,41]. For this analysis, the results of the HHS that were recorded during the last follow-up evaluation were used. In all studies but one [33], the mean HHS was higher in DM-THA patients. The overall mean difference was statistically significant (4.55, 95% CI: 2.45 to 6.65, *p* < 0.0001), with an *I*^2^ index of 71% (Figure 4).

(iv) Dislocation rate

**Figure 5:**
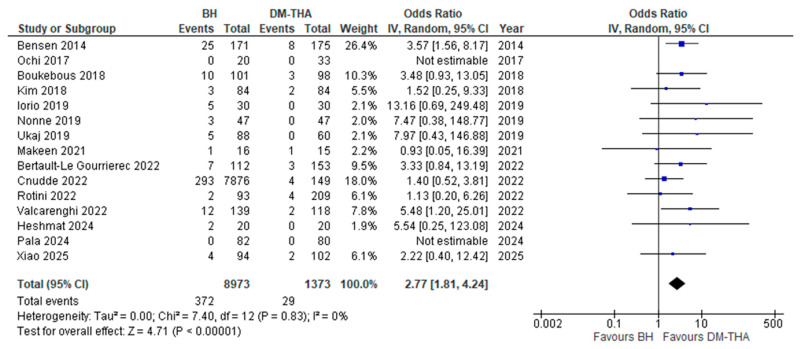
Forest plot for comparison of Dislocation Rate [26,27,28,29,31,32,33,34,35,36,37,38,39,40,41].

The comparative postoperative dislocation rates between groups were addressed in 15 studies [26,27,28,29,31,32,33,34,35,36,37,38,39,40,41]. For studies providing dislocation rates at various time points, the dislocation rate at the last follow-up was included in the analysis, except for one study with a very long follow-up period, where the dislocation rate at 2 years postoperatively was used instead [35]. In two studies, no dislocations occurred in either group [27,39]. Only one study with a small sample size demonstrated equal rates of dislocations between groups [34]; in the remaining 12 studies, the dislocation rate was higher in the BH group. In our meta-analysis, BH was associated with a statistically significant increase in risk of dislocation ([OR] 2.77, 95% CI: 1.81 to 4.24, *p* < 0.0001), with minimal heterogeneity (*I^2^* = 0%) (Figure 5).

(v) Reoperation rate

**Figure 6:**
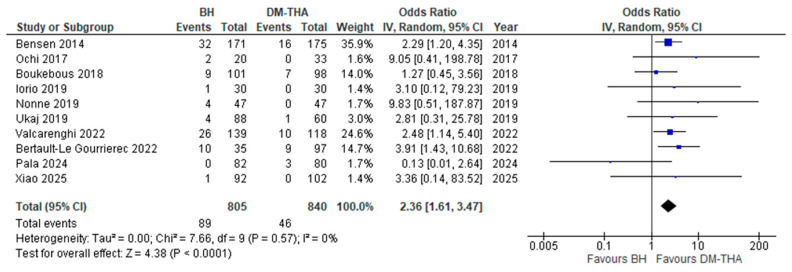
Forest plot for comparison of Reoperation Rate [26,27,28,31,32,33,35,38,39,41].

Ten studies examined the all-purpose reoperation rate during the follow-up period [26,27,28,31,32,33,35,38,39,41]. The most common causes for reoperation included dislocation, surgical site infection, periprosthetic fracture, and aseptic loosening. The study by Pala et al. found a higher reoperation rate in the DM-THA group [39]. In the remaining nine studies, patients who underwent BH were reoperated on at a higher rate. The odds ratio for reoperation in the BH group was estimated at ([OR] 2.36, 95% CI: 1.61 to 3.47, *p* < 0.0001) and was statistically significant, with minimal heterogeneity (*I*^2^ = 0%) (Figure 6).

(vi) Mortality rate

**Figure 7:**
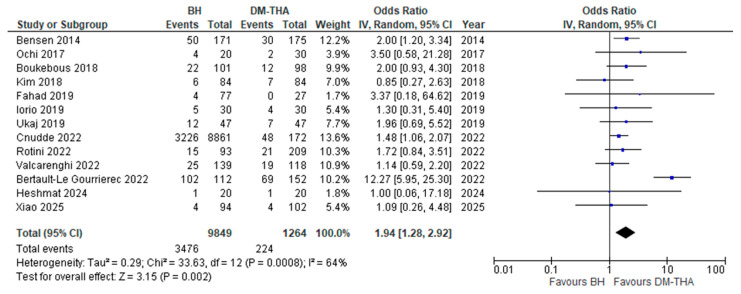
Forest plot for comparison of Mortality Rate [26,27,28,29,30,31,32,33,34,36,38,39,41].

Thirteen studies assessed the postoperative mortality rates for each treatment group [26,27,28,29,30,31,32,35,36,37,38,40,41]. Ten studies provided data on 1-year mortality [26,27,28,29,30,31,32,33,34,36,38,39,41]. For the remaining studies, the available mortality rates at 6 months, 2 years, and 5 years, respectively, after the operation were used in our analysis. A single study showed a higher mortality rate in the DM-THA group [29], and another presented equal rates between the groups [40]. The remaining 11 studies demonstrated higher postoperative mortality for BH patients. Overall, a statistically significant increase in mortality risk for the BH group was found ([OR] 1.94, 95% CI: 1.28 to 2.92, *p* < 0.0001). The *I*^2^ index was 62% (Figure 7).

## 4. Discussion

This systematic review and meta-analysis aimed to evaluate treatment outcomes between bipolar hip HA and DM-THA in patients with FNFs by analyzing all observational studies and randomised controlled trials in the literature that included and directly compared groups of patients treated with either of these two methods. Our results suggest that DM-THA provides superior outcomes in terms of postoperative functionality, pain, quality of life, and return to preoperative activity levels. Simultaneously, DM-THA presents a significantly reduced risk of long-term complications, including mortality. Conversely, it may be linked to higher perioperative morbidity due to the prolonged duration of the operation and increased blood loss during surgery. Both factors and their consequences (i.e., blood transfusion, aggressive fluid management, delayed/impaired postoperative mobilisation) are well-recognised predictors of short-term complications, such as surgical site infection and several adverse medical events [42,43,44,45,46].

It is thus apparent that the benefits of each treatment method do not always outweigh their risks and vice versa. This fact raises the question of whether these patients could be stratified based on specific criteria that would enable patient-tailored treatment. Age and pre-injury activity level are usually the most important parameters determining allocation to a treatment method in most institutions worldwide [47,48]. Traditionally, conventional THA has been the preferred treatment for younger active patients, and the age threshold for THA has increased in recent years [49,50]. However, conventional THA has been linked to a higher dislocation rate compared to HA, even in patients with similar characteristics [20,51,52,53]. On the contrary, not only has DM-THA shown a lower dislocation rate than conventional THA in the treatment of FNFs [15,54], but it has also, according to our study, demonstrated a significantly lower risk of dislocation compared to bipolar HA. This finding is notable because DM-THA could potentially bridge a “treatment gap” in patient groups where conventional THA would significantly raise the risk of dislocation, but HA would impede daily activities and diminish quality of life.

Another key factor to consider when deciding which procedure to choose is the presence of cognitive impairment or neuromuscular disease. These individuals are characterised by compromised stability and walking ability, making them prone to postoperative delirium, all of which are associated with an increased risk of falls that could lead to dislocation, peri-prosthetic fracture, and higher mortality [55,56]. Three of the studies included in our analysis (two RCTs and one registry-based retrospective cohort study) were conducted exclusively on patients suffering from cognitive impairment without neuromuscular deficit [31] or a combination of patients diagnosed with either cognitive impairment or a neuromuscular disorder [33,36]. All studies reported similar or favourable outcomes for DM-THA concerning dislocation, reoperation, and mortality rates. These results further enhance the status of DM-THA as a viable alternative to HA in this frail group of patients [57]. 

Individual anatomical characteristics of each patient represent another potential deciding factor in opting for DM-THA over HA. Acetabular deformities, such as a shallow acetabular socket or posterior wall deficiency, have been associated with a significantly increased risk of dislocation following HA [58,59]. Because these dislocations stem from the patient’s inherent hip morphology, they are often recurrent and typically require revision surgery. Additionally, other severe complications that may necessitate reoperation, such as acetabular prosthetic protrusion [60], can also occur. Therefore, DM-THA, by better replicating normal hip anatomy, could offer a safer option for most patients with dysplastic hips. DM-THA should also be considered for patients with preexisting osteoarthritis due to the risk of erosion of the acetabular cartilage and the consequent need for revision [61]. However, it is important to note that in the studies included in our review, revision surgery for the aforementioned cause in HA patients was an exceedingly rare complication, likely due to the short life expectancy of these patients, which suggests that THA is not a significant improvement in that aspect for older individuals.

As previously mentioned, a valid concern with DM-THA is the prolonged duration of surgery and the higher volume of blood loss. This is to be expected because DM-THA involves the additional surgical steps of preparation of the acetabular socket and insertion of the acetabular component. Nevertheless, there were discrepancies in our meta-analysis regarding these parameters. In some studies, the mean differences in operative time [26,32,39,41] and estimated blood loss [32,41] were minimal and likely without clinical significance. This observation suggests that these variables largely depend on the surgical team and could be minimised. Admittedly, most operations on hip fracture patients worldwide are carried out by orthopaedic surgeons without specialised training in hip arthroplasty. Hip HA has also been established as the standard of care for decades, before the surge of THA in recent years. Hence, most orthopaedic surgeons handle a considerably higher volume of HAs and are more familiar with them than THA, particularly DM-THA [62]. Therefore, surgeons involved in these procedures must undergo rigorous training in the assessment and placement of the acetabular component, as well as the proper handling of periacetabular soft tissues. Preoperative templating, educating the entire operating room staff, and cooperation with the anesthesiologists could further mitigate these issues. In any case, DM-THA could be a very beneficial solution with negligible risks when performed by a skilled and experienced surgeon.

A previous systematic review and meta-analysis on comparative outcomes of BH and DM-THA was conducted in 2021 by Ma et al. [19], exhibiting similar results to those of our research for all outcomes. Age, female sex, posterolateral surgical approach, and implant choice showed no link to dislocation or reoperation [19]. The addition of eight newer studies [34,35,36,37,38,39,40,41], including two RCTs [34,40], enhances the consistency and reliability of these findings to a greater extent.

Our study had limitations. Although this was the largest systematic review and meta-analysis to date, with studies that directly compared outcomes of BH and DM-THA, only four [31,32,34,40] out of 16 studies were randomised controlled trials, which were evaluated at moderate [31,40] or high risk [32,34] of bias according to the ROB2 assessment tool. The remaining 12 [26,27,28,29,30,33,35,36,37,38,39,41] were observational studies, and they contributed the majority of patients to our analysis. Indeed, most of these studies comprised highly heterogeneous groups of patients. Furthermore, the meta-analyses on certain outcomes were characterised by significant heterogeneity. The average age of BH patients was only 2.8 years higher than that of DM-THA patients across all studies, but these patients were likely still subject to selection bias by possibly being assigned to a treatment method based on their overall health profile and preinjury activity levels. As previously noted, the rationale for allocation to treatment groups also varied considerably among studies. It should be remarked, however, that in several of the retrospective studies, patients were not allocated to a treatment method based on patient-related factors, such as age and comorbidities, but rather according to different institutional protocols for separate time periods, or in the case of a multicentre study, varying treatment protocols between institutions during the same period. This process introduced a form of quasi-randomisation in patient selection, thereby reducing overall selection bias. Other variables, such as surgical approach and technique, fixation method, varying implants, varying follow-up periods and differing surgeons, even within the same study, all represent significant confounding factors that could influence our results substantially and contribute to the high heterogeneity that was observed in several meta-analyses. Moreover, the inclusion of studies focusing on special subgroups of patients (i.e., cognitively impaired and neuromuscular deficient individuals) alongside studies that consisted solely of self-sufficient patients and others that did not address these concerns at all further reduces the generalisability of our findings. Additionally, the meta-analysis for reoperation rate, despite displaying overwhelmingly favourable results for DM-THA without heterogeneity, did not distinguish between minor and major causes and subsequent reoperation procedures.

## 5. Conclusions

Our study indicates that DM-THA for the treatment of FNFs offers a reliable and safe alternative to BH, providing better functional results and a lower risk of long-term complications in certain subgroups of patients. However, current evidence is characterised by substantial limitations, rendering it insufficient to recommend the procedure for specific groups. Thus, there are no practical clinical criteria provided to guide when one procedure should be preferred over the other. More RCTs and propensity-score-matched observational studies, involving a larger number of patients and separate groups by age, mental status, and functionality, should be conducted to draw more robust conclusions. Nonetheless, in modern clinical practice, DM-THA should be taken into consideration as a potential option for the treatment of FNF patients after carefully accounting for their individual characteristics.

## Figures and Tables

**Table 1:** Study design of the included studies and patients’ demographics.

Authors	Year, Country	Study Type	Total Number of Patients (BH/DM)	Time of Surgery	Age at Surgery * (Years) (BH/DM)	Follow up * (Months) (BH/DM)	Sex (BH/DM) (Men–Women)	Studied Outcomes
Bensen et al [26].	2014, Denmark	RCS	346	2007–2010	84.1/75.9	25.3/21.7	40–131/52–123	BL, Disl, R & M
			(171/175)					
Ochi et al. [27]	2017, Japan	RCS	53	2009–2015	80/75.4	28.2/15.8	4–16/7–26	BL, Disl, R & M
			(20/33)					
Boukebous et al. [28]	2018, France	RCS	199	2010–2015	83.3/77.8	25.8/24.2	28–73/28–70	Disl, R & M
			(101/98)					
Kim et al. [29]	2018, South Korea	RCS	168	2007–2016	72.9/73.1	22.1/21.7	27–57/26–58	BL, Disl & M, HHS
			(84/84)					
Fahad et al. [30]	2019, Pakistan	RCS	104	2015–2017	71.1/69.3	20.6/10.9	31–46/13–14	M, HHS
			(77/27)					
Iorio et al. [31]	2019, Italy	RCT	60	2015–2017	83/82	12/12	13–17/12–18	Disl, R & M
			(30/30)					
Ukaj et al. [32]	2019, Kosovo	RCT	94	2008–2014	77.6/78.1	36/36	32–15/23–24	BL, Disl, R & M, HHS
			(47/47)					
Nonne et al. [33]	2019, Italy	RCS	148	2013–2017	86.1/87.6	28.3	22–66/15–45	Disl & R
			(88/60)					
Makeen et al. [34]	2021, Egypt	RCT	33	2018–2020	71.1/70.4	24	10–7/7–9	BL, HHS Disl
			(17/16)					
Bertault-Le Gourrierec et al. [35]	2022, France	RCS	265	2010–2013	85.4/78.5	62	28–84/42–111	Disl, R & M
			(112/153)					
Cnudde et al. [36]	2022, Sweeden	RCS	9033	2005–2014	83.3/77.9	≥36	2941–5920/70–102	Disl, R & M
			(8861/172)					
Rotini et al. [37]	2022, Italy	RCS	302	2018–2019	87/84.8	≥6	31–62/54–155	Disl & M
			(93/209)					
Valcarenghi et al. [38]	2022, Belgium	RCS	257	2015–2019	85/81	17.6/18.1	26–113/21–97	Disl, R & M
			(139/118)					
Pala et al. [39]	2024, Italy	RCS	162	2020–2022	78/74	10.3/14.5	20–62/19–61	BL, HHS Disl &R
			(82/80)					
Heshmat et al. [40]	2024, Egypt	RCT	40	2018–2021	72.5/69	≥24	9–11/5–15	BL, HHS Disl & M
			(20/20)					
Xiao et al. [41]	2025, China	RCS	196	2017–2021	69.1/68.7	≥36	42–52/43–59	BL, HHS Disl, R & M
			(94/102)					

* Mean, BH: Bipolar Hip Hemiarthroplasty, DM: Dual Mobility Total Hip Arthroplasty, RCS: Retrospective Comparative Study, HHS: Harris Hip Score, RCT: Randomised Controlled Trial, BL: Blood Loss, Disl: Dislocation, R: Reoperation. M: Mortality rate.

**Table 2:** Comparative outcomes between BH and DM in the included studies.

Authors	Outcomes				
	Estimated Blood Loss * (mL)	Dislocation Rate **	Reoperation Rate **	Mortality Rate ***	HHS **
	BH–DM	BH–DM	BH–DM	BH–DM	BH–DM
Bensen et al. [26]	310–424	25/171–8/175	32/171–16/175	50/171–30/175	N/A
Ochi et al. [27]	148–480	0/20–0/33	2/20–0/33	4/20–2/30	N/A
Boukebous et al. [28]	N/A	10/101–3/98	9/101–7/98	22/101–12/98	N/A
Kim et al. [29]	489–634	3/84–2/84	N/A	6/84–7/84	79.3–83.4
Fahad et al. [30]	N/A	N/A	N/A	4/77–0/27	68.8–76.8
Iorio et al. [31]	N/A	5/30–0/30	1/30–0/30	5/30–4/30	N/A
Ukaj et al. [32]	159–178	3/47–0/47	4/47–0/47	12/47–7/47	88.3–92.5
Nonne et al. [33]	N/A	5/88–0/60	4/88–1/60	N/A	81.7–79.8
Makeen et al. [34]	365–528	1/16–1/15	N/A	N/A	70.4–77.9
Bertault-Le Gourrierec et al. [35]	N/A	7/112–3/153	10/35–9/97	102/112–69/152 ^a^	N/A
Cnudde et al. [36]	N/A	293/7876–4/149	N/A	36.4–27.9%	N/A
Rotini et al. [37]	N/A	2/93–4/209	N/A	15/93–21/209 ^b^	N/A
Valcarenghi et al. [38]	N/A	12/139–2/118	26/139–10/118	25/139–19/118	N/A
Pala et al. [39]	N/A	0/82–0/80	0/82–3/80	N/A	81–87.5
Heshmat et al. [40]	700–430	2/20–0/–	N/A	1/20–1/20 ^c^	88–92.5
Xiao et al. [41]	257–285	4/94–2/102	1/92–0/102	4/94–4/102	85–89.5

* Mean. ** At the last follow-up. *** At 1 year post-operatively. ^a^ at five years follow-up, ^b^ at six months follow-up, ^c^ at two years follow-up. BH: Bipolar Hemiarthroplasty, DM: Dual Mobility Total Hip Arthroplasty, Hgb: Haemoglobin, HHS: Harris Hip Score, N/A: Not Available.

**Table 3:** Surgical characteristics of the included studies.

Author	Surgical Approach (BH/DM)	Operative Time * (minutes) (BH/DM)	Fixation Type ** (%) (BH/DM)	Type of Implants (BH/DM)	Other Technical Considerations	Criteria for Allocation to Either BH, DM
Bensen et al. [26]	PL	68/74	US: 94.4/94.6 UC: 98.3	Stems: Corail/Corail, ANCA-FIT™, Cups: Saturne	PC and SER repair, Limited DM previous experience, MS	Preexisting OA: (DM), SP, Time period: (2007–8: BH, 2009–10:DM)
Ochi et al. [27]	DAA	89.4/108.3	US: 100 UC: 100	Stems: Profemur TL, AccoladeTMZF, TriLock Cups: Trident, MDM	Fluoroscopy use, additional cups screws in osteoporotic bone, MS	Time period: (2009–13: BH, 2013–15: DM)
Boukebous et al. [28]	PL	85/102	US: 22/ 55 UC: 70	Stems: Sem3 (CS), Louxor (UC), Cups: Galliléa (CC), Evora (UC)	MS	Time period: (2010–13: BHA, 2013–15: DM)
Kim et al. [29]	PL	64.2/77.3	US: 100 UC: 100	Stems: Accolade TMZF Cups: Trident PSL MDM	PC and SER repair, fluoroscopy use, SS	Time period: (2007–13: BH 2013–16: DM)
Fahad et al. [30]	DL, PL	N/A	US: 100 UC: 100	Stems: porous coated Cups: titanium alloy with HY coating	Cups stabilisation with two pegs and a screw, MS	N/A
Iorio et al. [31]	DL	48/59	US: 100 UC: 100	Stems: Excia/Pavi Cups: Quattro	N/A	-Randomisation
Ukaj et al. [32]	PL	57.8/63.7	US: 34.4 UC: 100	Stems: Pavi Cups: Quattro	PC repair, Cups stabilisation with six fins & four spikes, SS	-Randomisation
Nonne et al. [33]	PL	N/A	US: 85.2 UC: 96.7	Stems: S-Taper, Korus Cups: Dualis	N/A	N/A
Makeen et al. [34]	PL	66/95	US: N/A UC: 100	Stems: Exception, CPT/Exception Cups: Serf NOVAE Evolution	-PC and SER repair	-Randomisation
Bertault-Le Gourrierec et al. [35]	PL	N/A	US: 25 /85 UC: 92	Stems: N/A/ Cups: Liberty ATF, Novae SERF, others	MS	SP, Patient factors
Cnudde et al. [36]	DL:65.3%, PL:34.7%/ DL:40.1%, PL:59.9%	N/A	US: 0 UC: N/A	N/A	MS and institutions	Institution dependent
Rotini et al. [37]	PL	66.3/78.6	Both US & CS, (%N/A) Both UC & CC, (%N/A)	Stems: CCA, MS-30, SL/PolarStem, Taperloc, Hype, Apta Fix, Exeter, ABG, Accolade, Apta Cups: PolarCups, Avantage, Novae Stick, Trident, Novae	MS and institutions	SP, Patient factors
Valcarenghi et al. [38]	PL	N/A	US: most cases, (%N/A) UC: 100	N/A	PC and SER repair, MS	Preexisting OA: (DM-THA) SP
Pala et al. [39]	DA:4.9%, PL:3.6%, AL: 91.5%/ DA: 27.5%, PL:12.5%, AL:60%	85/101	US: 26.8 UC: 100	Stems: H-max, CL-trauma /Hype Cups: NOVAE SunFit	MS	Patient factors
Heshmat et al. [40]	DL	54.3/81.6	US: 0 UC: 40	Stems: N/A Cups: Avantage	MS	-Randomisation
Xiao et al. [41]	N/A	76.8/86.4	N/A	N/A	N/A	N/A

* Mean, ** Results are given as percentages of uncemented implants; the rest are cemented, BH: Bipolar Hip Hemiarthroplasty, DM: Dual Mobility Total Hip Arthroplasty, US: Uncemented Stems, UC: Uncemented Cups, CS: Cemented Stems, CC: Cemented Cups, SER: Short External Rotators, OA: Osteoarthritis, SOC: Standard of Care, DA: Direct Anterior approach, DL: Direct Lateral approach, PL: Posterolateral approach, AL: Anterolateral approach, HY: hydroxyapatite, PC: posterior capsule, N/A: Not Available, MS: multiple surgeons, SS: Single surgeon, SP: Surgeon preference.

**Table 4:** Cognitive and neuromuscular status of patients in the included studies.

Author	Cognitive Impairment % (BH/DM)	Neuromuscular Deficit % (BH/DM)
Bensen et al. [26]	N/A	N/A
Ochi et al. [27]	N/A	10/24.2
Boukebous et al. [28]	50/25	N/A
Kim et al. [29]	0 *	0 *
Fahad et al. [30]	N/A	N/A
Iorio et al. [31]	100 **	0 **
Ukaj et al. [32]	0	0
Nonne et al. [33]	100 ***	
Makeen et al. [34]	N/A	N/A
Bertault-Le Gourrierec et al. [35]	27.2	5.8
Cnudde et al. [36]	100 ***	
Rotini et al. [37]	N/A	N/A
Valcarenghi et al. [38]	32/40	N/A
Pala et al. [39]	0 *	0 *
Heshmat et al. [40]	N/A	N/A
Xiao et al. [41]	0 *	0 *

* Patients with either cognitive impairment or a neuromuscular disorder were excluded from the study. ** Only patients with cognitive impairment but without a neuromuscular disorder were included in the study. *** Only patients with either cognitive impairment or a neuromuscular disorder were included in the study. BH: Bipolar Hip Hemiarthroplasty, DM: Dual Mobility Total Hip Arthroplasty, N/A: Not Available.

## Data Availability

The dataset is available upon request from the authors.

## Supplementary Materials

The following supporting information can be downloaded at: https://www.mdpi.com/article/10.3390/jcm14145076/s1, Supplementary Material S1: The PRISMA 2020 Checklist, Supplementary Material S2: The search string used in our study, Supplementary Material S3: Quality assessment for randomised control trials included, based on the Cochrane Risk of Bias Assessment 2 Tool (RoB 2), Supplementary Material S4: Quality assessment for cohort studies included, based on the Newcastle-Ottawa-Scale (NOS). Supplementary Material S5. Studies excluded after full-text assessment; Supplementary Material S6. Implant companies in the included studies.

## Abbreviations

The following abbreviations are used in this manuscript:

FNF	Femoral Neck Fracture
HA	Hip Hemiarthroplasty
THA	Total Hip Arthroplasty
DM	Dual Mobility
BH	Bipolar Hip Hemiarthroplasty
DM-THA	Dual Mobility Total Hip Arthroplasty
PROMs	Patient Reported Outcome Measures
RCT	Randomised Controlled Trials
RoB2	Cochrane Risk of Bias 2 assessment tool
NOS	Newcastle-Ottawa Scale
CI	Confidence Interval
OR	Odds Ratio
HHS	Harris Hip Score
