# Supplementary material for: Are There Benefits of Total Hip Arthroplasty with Dual-Mobility Cups Compared to Bipolar Hemiarthroplasty for Femoral Neck Fractures in the Geriatric Population? A Systematic Review and Meta-Analysis of Comparative Studies
Source: J Clin Med. 2025 Jul 17;14(14):5076. doi: 10.3390/jcm14145076 (PMC12295041; doi:10.3390/jcm14145076)
Supplement: Supplementary file 1 [file jcm-14-05076-s001.zip › jcm-3748738-supplementary.pdf]

SUPPLEMENTARY MATERIAL

Supplementary Material S1. PRISMA Checklist

Section and Topic	Item #	Checklist item	Page where item is reported
**TITLE**			
Title	1	Identify the report as a systematic review.	1
**ABSTRACT**			
Abstract	2	See the PRISMA 2020 for Abstracts checklist.	1
**INTRODUCTION**			
Rationale	3	Describe the rationale for the review in the context of existing knowledge.	2
Objectives	4	Provide an explicit statement of the objective(s) or question(s) the review addresses.	2
**METHODS**			
Eligibility criteria	5	Specify the inclusion and exclusion criteria for the review and how studies were grouped for the syntheses.	3,4
Information sources	6	Specify all databases, registers, websites, organisations, reference lists and other sources searched or consulted to identify studies. Specify the date when each source was last searched or consulted.	3
Search strategy	7	Present the full search strategies for all databases, registers and websites, including any filters and limits used.	Supplementary Material
Selection process	8	Specify the methods used to decide whether a study met the inclusion criteria of the review, including how many reviewers screened each record and each report retrieved, whether they worked independently, and if applicable, details of automation tools used in the process.	3
Data collection process	9	Specify the methods used to collect data from reports, including how many reviewers collected data from each report, whether they worked independently, any processes for obtaining or confirming data from study investigators, and if applicable, details of automation tools used in the process.	3
Data items	10a	List and define all outcomes for which data were sought. Specify whether all results that were compatible with each outcome domain in each study were sought (e.g. for all measures, time points, analyses), and if not, the methods used to decide which results to collect.	3, 4, 5
	10b	List and define all other variables for which data were sought (e.g. participant and intervention characteristics, funding sources). Describe any assumptions made about any missing or unclear information.	3, 4, 5
Study risk of bias assessment	11	Specify the methods used to assess risk of bias in the included studies, including details of the tool(s) used, how many reviewers assessed each study and whether they worked independently, and if applicable, details of automation tools used in the process.	3, Supplementary Material
Effect measures	12	Specify for each outcome the effect measure(s) (e.g. risk ratio, mean difference) used in the synthesis or presentation of results.	4
Synthesis methods	13a	Describe the processes used to decide which studies were eligible for each synthesis (e.g. tabulating the study intervention characteristics and comparing against the planned groups for each synthesis (item #5)).	4, 5
	13b	Describe any methods required to prepare the data for presentation or synthesis, such as handling of missing summary statistics, or data conversions.	4
	13c	Describe any methods used to tabulate or visually display results of individual studies and syntheses.	N/A
	13d	Describe any methods used to synthesize results and provide a	4

Supplementary Material S1. PRISMA Checklist

Section and Topic	Item #	Checklist item	Page where item is reported
		rationale for the choice(s). If meta-analysis was performed, describe the model(s), method(s) to identify the presence and extent of statistical heterogeneity, and software package(s) used.	
	13e	Describe any methods used to explore possible causes of heterogeneity among study results (e.g. subgroup analysis, meta-regression).	N/A
	13f	Describe any sensitivity analyses conducted to assess robustness of the synthesized results.	N/A
Reporting bias assessment	14	Describe any methods used to assess risk of bias due to missing results in a synthesis (arising from reporting biases).	N/A
Certainty assessment	15	Describe any methods used to assess certainty (or confidence) in the body of evidence for an outcome.	N/A
**RESULTS**			
Study selection	16a	Describe the results of the search and selection process, from the number of records identified in the search to the number of studies included in the review, ideally using a flow diagram.	5, Figure 1
	16b	Cite studies that might appear to meet the inclusion criteria, but which were excluded, and explain why they were excluded.	Figure 1, Supplementary Material
Study characteristics	17	Cite each included study and present its characteristics.	5,6
Risk of bias in studies	18	Present assessments of risk of bias for each included study.	Supplementary Material
Results of individual studies	19	For all outcomes, present, for each study: (a) summary statistics for each group (where appropriate) and (b) an effect estimate and its precision (e.g. confidence/credible interval), ideally using structured tables or plots.	Table 1, Table 2
Results of syntheses	20a	For each synthesis, briefly summarise the characteristics and risk of bias among contributing studies.	6, 7, 10
	20b	Present results of all statistical syntheses conducted. If meta-analysis was done, present for each the summary estimate and its precision (e.g. confidence/credible interval) and measures of statistical heterogeneity. If comparing groups, describe the direction of the effect.	10-13, Figures 2-7
	20c	Present results of all investigations of possible causes of heterogeneity among study results.	N/A
	20d	Present results of all sensitivity analyses conducted to assess the robustness of the synthesized results.	N/A
Reporting biases	21	Present assessments of risk of bias due to missing results (arising from reporting biases) for each synthesis assessed.	N/A
Certainty of evidence	22	Present assessments of certainty (or confidence) in the body of evidence for each outcome assessed.	N/A
**DISCUSSION**			
Discussion	23a	Provide a general interpretation of the results in the context of other evidence.	14,15
	23b	Discuss any limitations of the evidence included in the review.	15
	23c	Discuss any limitations of the review processes used.	15
	23d	Discuss implications of the results for practice, policy, and future research.	15
**OTHER INFORMATION**			
Registration	24a	Provide registration information for the review, including register	3

Supplementary Material S1. PRISMA Checklist

Section and Topic	Item #	Checklist item	Page where item is reported
and protocol		name and registration number, or state that the review was not registered.	
	24b	Indicate where the review protocol can be accessed, or state that a protocol was not prepared.	N/A
	24c	Describe and explain any amendments to information provided at registration or in the protocol.	N/A
Support	25	Describe sources of financial or non-financial support for the review, and the role of the funders or sponsors in the review.	N/A
Competing interests	26	Declare any competing interests of review authors.	16
Availability of data, code and other materials	27	Report which of the following are publicly available and where they can be found: template data collection forms; data extracted from included studies; data used for all analyses; analytic code; any other materials used in the review.	Tables and Supplementary Material

From: Page MJ, McKenzie JE, Bossuyt PM, Boutron I, Hoffmann TC, Mulrow CD, et al. The PRISMA 2020 statement: an updated guideline for reporting systematic reviews. BMJ 2021;372:n71. doi: 10.1136/bmj.n71. This work is licensed under CC BY 4.0. To view a copy of this license, visit https://creativecommons.org/licenses/by/4.0/

Supplementary Material S2. Search Strategies

<u>Pubmed search string:</u>

1. *(((((dual mobility [Title/Abstract]) OR (DM[Title/Abstract])) OR (Tripolar [Title/Abstract])) OR (acetabular component [Title/Abstract])) OR (cup [Title/Abstract])) OR ("Arthroplasty, Replacement, Hip"[Mesh])*
2. *(((("Hemiarthroplasty"[Mesh]) OR (bipolar [Title/Abstract])) OR (hemiarthroplasty*[Title/Abstract])) OR (partial hip [Title/Abstract])) OR (hemi [Title/Abstract])*
3. *((((((("Hip Fractures"[Mesh])) OR (intracapsular fracture*[Title])) OR (hip fracture*[Title])) OR (neck fracture*[Title])) OR (non-elective [Title])) OR (femoral [Title])) OR (femur [Title]))*
4. *(((((((comparative [Title/Abstract]) OR (comparison [Title/Abstract])) OR (outcome*[Title/Abstract])) OR (risk [Title/Abstract])) OR (survival [Title/Abstract])) OR (assessment [Title/Abstract])) OR ("Treatment Outcome"[Mesh])) OR ("Treatment Expectations"[Mesh])*
5. *#1 AND #2 AND #3 AND #4*

<u>Cochrane Library search string</u>

#1 ((dual NEXT mobility) OR (DM) OR (tripolar) OR (acetabular NEXT component) OR (cup)):ti,ab,kw (Word variations have been searched)

#2 MeSH descriptor: [Arthroplasty, Replacement, Hip] explode all trees

#3 ((bipolar) OR (hemiarthroplast) OR (partial NEXT hip) OR (hemi)):ti,ab,kw*

#4 ((intracapsular NEXT fracture) OR (hip NEXT fracture*) OR (neck NEXT fracture*) OR (non-elective) OR (femoral) OR (femur)):ti*

#5 MeSH descriptor: [Hip Fractures] explode all trees

#6 ((comparative) OR (comparison) OR (outcomes) OR (risk) OR (survival) OR (assessment)):ti,ab,kw*

#7 MeSH descriptor: [Treatment Outcome] explode all trees

#8 #1 OR #2

#9 #4 OR #5

#10 #6 OR #7

#11 #3 AND #8 AND #9 AND #10

<u>ScienceDirect search string:</u>

("Dual Mobility" OR DM) AND (bipolar OR hemiarthroplasty) AND (“intracapsular fracture” OR “hip fracture” OR "neck fracture" OR femoral OR femur)

Supplementary Material S3. Quality assessment for randomized control trials included, based on the Cochrane Risk of Bias Assessment 2 Tool (RoB 2).

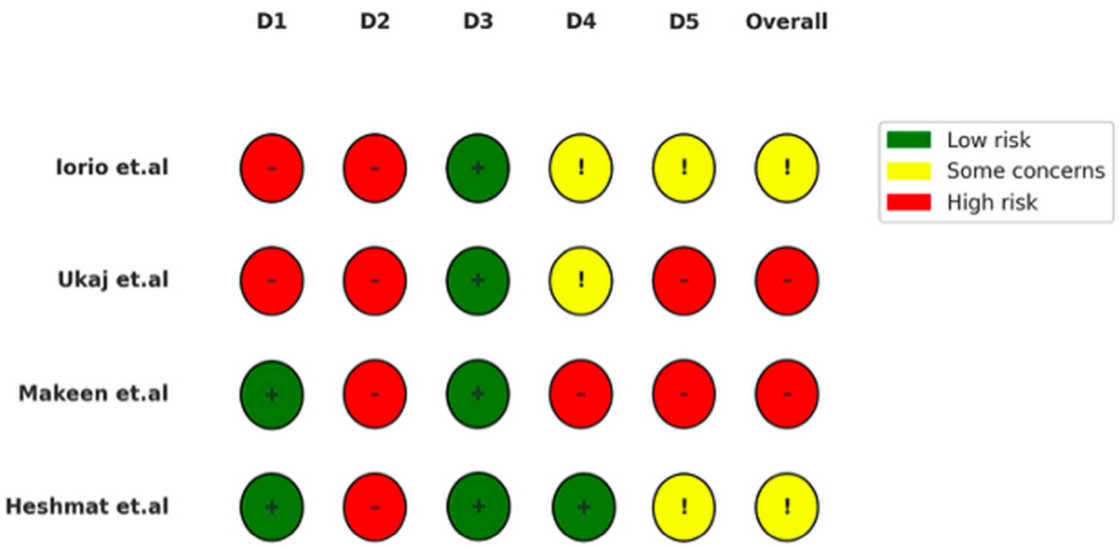

D1: Randomisation process
D2: Deviations from intended interventions
D3: Missing outcome data
D4: Measurement of the outcome
D5: Selection of the reported result

Supplementary Material S4. Quality assessment for cohort studies included, based on the Newcastle-Ottawa-Scale (NOS).

Author	Year	Selection Bias Assessment (Maximum 4 stars)				Comparability (Maximum 2 stars)	Outcome (Maximum 3 stars)			Total score (Maximum 9 stars)
		Representativeness of the exposed cohort	Selection of the non-exposed cohort	Ascertainment of exposure	Outcome not present at start	Design or analysis controls for confounding	Assessment of outcome	Follow-up long enough	Adequacy of follow-up	
Bensen et al.	2014	1	1	1	1	1	1	1	1	8
Ochi et al.	2017	1	1	1	1	2	1	1	1	9
Boukebous et al.	2018	1	1	0	1	0	1	1	1	6
Kim et al.	2018	1	1	1	1	2	1	1	1	9
Fahad et al.	2019	1	1	1	1	1	1	1	1	8
Nonne et al.	2019	1	1	1	1	1	1	1	1	8
Bertault et al.	2022	1	1	0	1	0	1	1	1	6
Cnudde et al.	2022	1	1	1	1	1	1	1	0	7
Rotini et al.	2022	1	1	1	1	1	1	1	0	7
Valcarenghi et al.	2022	1	1	1	1	1	1	1	1	9
Pala et al.	2024	0	1	1	1	1	1	0	1	6
Xiao et al.	2025	1	1	1	1	1	1	1	1	8

Supplementary Material S5. Studies excluded after full-text assessment

Studies excluded after full-text assessment			Reason for exclusion
Year	Authors	Title	
2025	Yin et al	Comparison of the surgical efficacy of total hip replacement versus hemiarthroplasty in the treatment of femoral neck fractures in elderly patients with sarcopenia DOI: 10.1371/journal.pone.0321070	Conventional THA only, no DM subgroup
2025	Uzel et al	Comparison of Hemiarthroplasty, total hip arthroplasty, and internal fixation for hip fractures in patients over eighty years of age: factors affecting mortality: a nationwide cohort study of fifty three thousand, four hundred and ninety five patients from Türkiye DOI: 10.1007/s00264-025-06412-8	Conventional THA only, no DM subgroup
2025	Demirel et al	Epidemiology, treatment, and mortality of femoral neck fractures in patients over the age of 65 years: a nationwide retrospective cohort study of 83,789 cases in Turkey DOI: 10.1177/11207000241312887	Conventional THA only, no DM subgroup
2025	Wang et al	Comparing surgical readmission, in-hospital complications, and charges between total hip arthroplasty and hemiarthroplasty for geriatric femoral neck fractures DOI: 10.1302/0301-620X.106B12.BJJ-2024-0321.R1	Conventional THA only, no DM subgroup
2024	Hatano et al	Increased early complications after total hip arthroplasty compared with hemiarthroplasty in older adults with a femoral neck fracture DOI: 10.1302/0301-620X.106B9.BJJ-2024-0089.R1	Conventional THA only, no DM subgroup
2024	Mori et al	Does total hip arthroplasty in elderly patients with femoral neck fractures reduce complications? A Japanese DPC study DOI: 10.1016/j.jos.2024.06.011	Conventional THA only, no DM subgroup
2024	Kim et al	Comparative Interrupted Time Series Analysis of Direct Medical Expense and Length of Stay in Elderly Patients with Femoral Neck Fractures Who Underwent Total Hip Arthroplasty and Hemiarthroplasty: A Real World Nationwide Database Study DOI: 10.4055/cios23282	Conventional THA only, no DM subgroup
2023	El-Deeb et al	Dual Mobility Cup in Fractures of the Femoral Neck in Neuromuscular Disorders and Cognitive Dysfunction Patients above 60 years-old DOI: 10.22038/ABJS.2023.65924.3157	DM-THA cohort only, no bipolar comparison group
2023	Elyahu et al	Better Short-term Outcomes after Total Hip Arthroplasty Compared to Hemiarthroplasty in Active Older Patients with Displaced Intracapsular Femoral Neck Fracture PMID: 38142319	Conventional THA only, no DM subgroup
2024	Okike et al	Unipolar Hemiarthroplasty, Bipolar Hemiarthroplasty, or Total Hip Arthroplasty for Hip Fracture in Older Individuals DOI: 10.2106/JBJS.23.00486	Conventional THA only, no DM subgroup
2023	Pangaud et al	Reduced mortality associated to cementless total hip arthroplasty in femoral neck fracture DOI: 10.1038/s41598-023-43790-8	Conventional THA only, no DM subgroup
2023	Luo et al	Total hip arthroplasty versus hemiarthroplasty in the treatment of active elderly patients over 75 years with displaced femoral neck fractures: a retrospective study DOI: 10.1186/s12891-023-06860-6	Conventional THA only, no DM subgroup
2023	Edelstein et al	Hemiarthroplasty Versus Total Hip Arthroplasty for Femoral Neck Fracture in Elderly Patients: Twelve-Month Risk of Revision and Dislocation in an Instrumental Variable Analysis of Medicare Data DOI: 10.2106/JBJS.23.00247	Conventional THA only, no DM subgroup

2023	Parker et al	A long term follow-up for a randomised trial of total hip arthroplasty versus hemiarthroplasty for displaced intracapsular fractures<br>DOI: 10.1016/j.injury.2023.110925	Conventional THA only, no DM subgroup
2023	Onggo et al	Comparing outcomes of total hip arthroplasty versus hemiarthroplasty in neck of femur fracture patients: an Australian registry study<br>DOI: 10.1007/s00068-023-02305-w	Conventional THA only, no DM subgroup
2023	Tohidi et al	Comparative Effectiveness of Total Hip Arthroplasty and Hemiarthroplasty for Femoral Neck Fracture: A Propensity-Score-Matched Cohort Study<br>DOI: 10.2106/JBJS.22.01193	Conventional THA only, no DM subgroup
2023	Atik & Hangody	Total hip replacement or hip hemiarthroplasty for the treatment of displaced femoral neck fractures in the elderly?<br>DOI: 10.52312/jdrs.2022.57908	Review Article
2022	Hoskins et al	Is the Revision Rate for Femoral Neck Fracture Lower for Total Hip Arthroplasty Than for Hemiarthroplasty?: A Comparison of Registry Data for Contemporary Surgical Options<br>DOI: 10.2106/JBJS.21.01256	Includes both a bipolar cohort and a mixed conventional/DM THA cohort, but doesn't differentiate between conventional and DM patients
2022	Kizkapan et al	Correlation between Harris, modified Harris hip, and Oxford hip scores of patients who underwent hip arthroplasty and hemiarthroplasty following hip fracture<br>DOI: 10.14744/tjtes.2020.74560	Conventional THA only, no DM subgroup
2022	Farey et al	Bipolar Hemiarthroplasty Does Not Result in a Higher Risk of Revision Compared with Total Hip Arthroplasty for Displaced Femoral Neck Fractures: An Instrumental Variable Analysis of 36,118 Procedures from the Australian Orthopaedic Association National Joint Replacement Registry<br>DOI: 10.2106/JBJS.21.00972	Conventional THA only, no DM subgroup
2022	DeKeyser et al	Increased Complications and Cost Associated With Hip Arthroplasty for Femoral Neck Fracture: Evaluation of 576,119 Medicare Patients Treated With Hip Arthroplasty<br>DOI: 10.1016/j.arth.2021.12.027	Conventional THA only, no DM subgroup
2022	Bordes et al	Outcomes and survival of a modern dual mobility cup and uncemented collared stem in displaced femoral neck fractures at a minimum 5-year follow-up<br>DOI: 10.1016/j.otsr.2021.103164	DM-THA cohort only, no bipolar comparison group
2022	Yoo et al	Comparison of Mortality, Length of Hospital Stay and Transfusion between Hemiarthroplasty and Total Hip Arthroplasty in Octo- and Nonagenarian Patients with Femoral Neck Fracture: a Nationwide Study in Korea<br>DOI: 10.3346/jkms.2021.36.e300	Conventional THA only, no DM subgroup
2022	Wang et al	Total hip arthroplasty or hemiarthroplasty for femoral neck fractures in elderly patients with neuromuscular imbalance<br>DOI: 10.1007/s40520-021-01976-y	Conventional THA only, no DM subgroup
2021	Hayden et al	No Difference Between Hemiarthroplasty and Total Hip Arthroplasty in the Treatment of Pathologic Femoral Neck Fractures<br>DOI: 10.1016/j.arth.2021.06.015	Conventional THA only, no DM subgroup
2022	Craig et al	Clinical Outcomes for Hemiarthroplasty Versus Total Hip Arthroplasty in Patients With Femoral Neck Fracture Who Meet Published National Criteria for Total Hip Arthroplasty<br>DOI: 10.1097/BOT.0000000000002143	Conventional THA only, no DM subgroup
2022	Pass et al	Differences of hemiarthroplasty and total hip replacement in orthogeriatric treated elderly patients: a retrospective analysis of the Registry for Geriatric Trauma DGU®<br>DOI: 10.1007/s00068-020-01559-y	Conventional THA only, no DM subgroup

2020	Viswanathan et al	Treatment of displaced intracapsular fractures of the femoral neck with total hip arthroplasty or hemiarthroplasty<br>DOI: 10.1302/0301-620X.102B6.BJJ-2019-1459.R1	Conventional THA only, no DM subgroup
2020	Suarez et al	Hemiarthroplasty vs Total Hip Arthroplasty for Femoral Neck Fractures: 2010-2017 Trends in Complication Rates<br>DOI: 10.1016/j.arth.2020.02.040	Conventional THA only, no DM subgroup
2021	Stucinskas et al	Bipolar hemiarthroplasty versus total hip arthroplasty in femoral neck fracture patients: results from Lithuanian Arthroplasty Register<br>DOI: 10.1177/1120700020907124	Conventional THA only, no DM subgroup
2020	Hansson et al	More hip complications after total hip arthroplasty than after hemi-arthroplasty as hip fracture treatment: analysis of 5,815 matched pairs in the Swedish Hip Arthroplasty Register<br>DOI: 10.1080/17453674.2019.1690339	Conventional THA only, no DM subgroup
2021	Lee et al	Hemiarthroplasty versus total hip arthroplasty for femoral neck fractures in patients with chronic obstructive pulmonary disease<br>DOI: 10.1007/s00068-019-01234-x	Conventional THA only, no DM subgroup
2019	Parker & Cawley	Treatment of the displaced intracapsular fracture for the 'fitter' elderly patients: A randomised trial of total hip arthroplasty versus hemiarthroplasty for 105 patients<br>DOI: 10.1016/j.injury.2019.09.018	Conventional THA only, no DM subgroup
2019	Varady et al	Short-Term Morbidity and Mortality After Hemiarthroplasty and Total Hip Arthroplasty for Pathologic Proximal Femur Fractures<br>DOI: 10.1016/j.arth.2019.06.019	Conventional THA only, no DM subgroup
2019	Lin et al	Comparison of Mid-term Survivorship and Clinical Outcomes between Bipolar Hemiarthroplasty and Total Hip Arthroplasty with Cementless Stem: A Multicenter Retrospective Study<br>DOI: 10.1111/os.12440	Conventional THA only, no DM subgroup
2019	Ravi et al	Comparing Complications and Costs of Total Hip Arthroplasty and Hemiarthroplasty for Femoral Neck Fractures: A Propensity Score-Matched, Population-Based Study<br>DOI: 10.2106/JBJS.18.00539	Conventional THA only, no DM subgroup
2019	Canton et al	Dual mobility total hip arthroplasty in the treatment of femoral neck fractures: a retrospective evaluation at mid-term follow-up<br>DOI: 10.23750/abm.v90i1-S.8070	DM-THA cohort only, no bipolar comparison group
2019	Jawad et al	Multi-state analysis of hemi- and total hip arthroplasty for hip fractures in the Swedish population-Results from a Swedish national database study of 38,912 patients<br>DOI: 10.1016/j.injury.2018.12.022	Conventional THA only, no DM subgroup
2019	Eskildsen et al	Age matters when comparing hemiarthroplasty and total hip arthroplasty for femoral neck fractures in Medicare patients<br>DOI: 10.1177/1120700018816924	Conventional THA only, no DM subgroup
2018	Nemes et al	Relative survival following hemi-and total hip arthroplasty for hip fractures in Sweden DOI: 10.1186/s12891-018-2321-2	Conventional THA only, no DM subgroup
2018	Moerman et al	Hemiarthroplasty and total hip arthroplasty in 30,830 patients with hip fractures: data from the Dutch Arthroplasty Register on revision and risk factors for revision<br>DOI: 10.1080/17453674.2018.1499069	Conventional THA only, no DM subgroup
2018	Barışhan et al	Comparison of hemiarthroplasty and total hip arthroplasty in elderly patients with displaced femoral neck fractures<br>DOI: 10.1177/0300060518770354	Conventional THA only, no DM subgroup
2018	Sonaje et al	Comparison of functional outcome of bipolar hip arthroplasty and total hip replacement in displaced femoral neck fractures in elderly in a developing country: a 2-year prospective study<br>DOI: 10.1007/s00590-017-2057-y	Conventional THA only, no DM subgroup

2017	Hansson et al	Reduced risk of reoperation after treatment of femoral neck fractures with total hip arthroplasty DOI: 10.1080/17453674.2017.1348095	Conventional THA only, no DM subgroup
2017	Quevedo et al	Patient survival and surgical re-intervention predictors for intracapsular hip fractures DOI: 10.1016/j.injury.2017.06.014	Conventional THA only, no DM subgroup
2017	Wang et al	Outcomes of Hemiarthroplasty and Total Hip Arthroplasty for Femoral Neck Fracture: A Medicare Cohort Study DOI: 10.1097/BOT.0000000000000814	Conventional THA only, no DM subgroup
2017	Nichols et al	Clinical Outcomes and 90-Day Costs Following Hemiarthroplasty or Total Hip Arthroplasty for Hip Fracture DOI: 10.1016/j.arth.2017.01.023	Conventional THA only, no DM subgroup
2017	Tol et al	Hemiarthroplasty or total hip arthroplasty for the treatment of a displaced intracapsular fracture in active elderly patients: 12-year follow-up of randomised trial DOI: 10.1302/0301-620X.99B2.BJJ-2016-0479.R1	Conventional THA only, no DM subgroup
2017	Uhler et al	Health Utility of Early Hemiarthroplasty vs Delayed Total Hip Arthroplasty for Displaced Femoral Neck Fracture in Elderly Patients: A Markov Model DOI: 10.1016/j.arth.2016.11.051	Conventional THA only, no DM subgroup
2017	Ercin et al	Risk factors for mortality in geriatric hip fractures: a compressional study of different surgical procedures in 785 consecutive patients DOI: 10.1007/s00590-016-1843-2	Conventional THA only, no DM subgroup
2016	Mariconda et al	Ambulatory Ability and Personal Independence After Hemiarthroplasty and Total Arthroplasty for Intracapsular Hip Fracture: A Prospective Comparative Study DOI: 10.1016/j.arth.2016.07.017	Conventional THA only, no DM subgroup
2016	Liodakis et al	Major Complications and Transfusion Rates After Hemiarthroplasty and Total Hip Arthroplasty for Femoral Neck Fractures DOI: 10.1016/j.arth.2016.02.019	Conventional THA only, no DM subgroup
2016	Burgers et al	Total medical costs of treating femoral neck fracture patients with hemi- or total hip arthroplasty: a cost analysis of a multicenter prospective study DOI: 10.1007/s00198-016-3484-z	Conventional THA only, no DM subgroup
2015	Sköldenberg et al	HOPE-trial: hemiarthroplasty compared to total hip arthroplasty for displaced femoral neck fractures in the elderly-elderly, a randomized controlled trial DOI: 10.1186/s12891-015-0763-3	Conventional THA only, no DM subgroup
2015	Jonas et al	Displaced intracapsular neck of femur fractures in the elderly: bipolar hemiarthroplasty may be the treatment of choice; a case control study DOI: 10.1016/j.injury.2015.06.047	Conventional THA only, no DM subgroup
2015	Muller et al	The fate of proximal femoral fractures in the 10th decade of life: an analysis of 117 consecutive patients DOI: 10.1016/j.injury.2015.06.048	Conventional THA only, no DM subgroup
2013	SooHoo et al	Comparison of complication rates between hemiarthroplasty and total hip arthroplasty for intracapsular hip fracture DOI: 10.3928/01477447-20130327-09	Conventional THA only, no DM subgroup
2013	Jameson et al	Cemented hemiarthroplasty or hip replacement for intracapsular neck of femur fracture? A comparison of 7732 matched patients using national data DOI: 10.1016/j.injury.2013.03.021	Conventional THA only, no DM subgroup
2012	Fisher et al	Open reduction internal fixation versus hemiarthroplasty versus total hip arthroplasty in the elderly: a review of the National Surgical Quality Improvement Program database DOI: 10.1016/j.jss.2012.07.004	Conventional THA only, no DM subgroup
2012	Fan et al	Comparison between bipolar hemiarthroplasty and total hip arthroplasty for unstable intertrochanteric fractures in elderly osteoporotic patients DOI: 10.1371/journal.pone.0039531	Conventional THA only, no DM subgroup

2012	Adam et al	Dual mobility cups hip arthroplasty as a treatment for displaced fracture of the femoral neck in the elderly. A prospective, systematic, multicenter study with specific focus on postoperative dislocation DOI: 10.1016/j.otsr.2012.01.005	Dm subgrouprt only, no bipolar comparison group
2012	Kannan et al	Arthroplasty options in femoral-neck fracture: answers from the national registries DOI: 10.1007/s00264-011-1354-z	Conventional THA only, no DM subgroup
2011	Avery et al	Total hip replacement and hemiarthroplasty in mobile, independent patients with a displaced intracapsular fracture of the femoral neck: a seven- to ten-year follow-up report of a prospective randomised controlled trial DOI: 10.1302/0301-620X.93B8.27132	Conventional THA only, no DM subgroup
2011	Hedbeck et al	Comparison of bipolar hemiarthroplasty with total hip arthroplasty for displaced femoral neck fractures: a concise four-year follow-up of a randomized trial DOI: 10.2106/JBJS.J.00474	Conventional THA only, no DM subgroup
2011	Xu et al	Comparison of effectiveness of three operations in treatment of displaced femoral neck fractures in the elderly patients PMID: 21261085	Full-text in a non-English language, no mention of a DM subgroup in abstract
2010	Van den Beckerom et al	A comparison of hemiarthroplasty with total hip replacement for displaced intracapsular fracture of the femoral neck: a randomised controlled multicentre trial in patients aged 70 years and over DOI: 10.1302/0301-620X.92B10.24899	Conventional THA only, no DM subgroup
2009	Slover et al	A cost-effectiveness analysis of the arthroplasty options for displaced femoral neck fractures in the active, healthy, elderly population DOI: 10.1016/j.arth.2008.05.008	Conventional THA only, no DM subgroup
2009	Macaulay et al	Prospective randomized clinical trial comparing hemiarthroplasty to total hip arthroplasty in the treatment of displaced femoral neck fractures: winner of the Dorr Award DOI: 10.1016/j.arth.2008.05.013	Conventional THA only, no DM subgroup
2007	Mouzopoulos et al	The four-year functional result after a displaced subcapital hip fracture treated with three different surgical options DOI: 10.1007/s00264-007-0321-1	Conventional THA only, no DM subgroup
2007	Blomfeldt et al	A randomised controlled trial comparing bipolar hemiarthroplasty with total hip replacement for displaced intracapsular fractures of the femoral neck in elderly patients DOI: 10.1302/0301-620X.89B2.18576	Conventional THA only, no DM subgroup
2007	Baker et al	Total hip arthroplasty and hemiarthroplasty in mobile, independent patients with a displaced intracapsular fracture of the femoral neck. A randomized, controlled trial DOI: 10.2106/JBJS.E.01373	Conventional THA only, no DM subgroup
2006	Keating et al	Randomized comparison of reduction and fixation, bipolar hemiarthroplasty, and total hip arthroplasty. Treatment of displaced intracapsular hip fractures in healthy older patients DOI: 10.2106/JBJS.E.00215	Conventional THA only, no DM subgroup
2005	Haentjens et al	Predictors of functional outcome following intracapsular hip fracture in elderly women. A one-year prospective cohort study DOI: 10.1016/j.injury.2005.02.002	Conventional THA only, no DM subgroup

2003	Schleicher et al	[Femoral neck fractures in the elderly -- bipolar hemiarthroplasty in total hip replacement] DOI: 10.1007/s00113-003-0597-6	Full-text in a non-English language, no mention of a DM subgroup in abstract
1999	Squires et al	Displaced intracapsular neck of femur fractures in mobile independent patients: total hip replacement or hemiarthroplasty? DOI: 10.1016/s0020-1383(99)00097-2	Conventional THA only, no DM subgroup
1999	Broos	Prosthetic replacement in the management of unstable femoral neck fractures in the elderly. Analysis of the mechanical complications noted in 778 fractures PMID: 10499394	Conventional THA only, no DM subgroup

Supplementary Material S6. Implant companies in the included studies

In order of listing in Table 2:

-DePuy, Orthopaedics, Inc., Warsaw, IN, USA; Wright Medical Technology, Inc., Arlington, TN, USA; Amplitude, Valence, France

-Wright Medical Technology, Memphis, TN, USA, Stryker Orthopaedics, Mahwah, NJ, USA, DePuy, Orthopaedics, Warsaw,IN

-SEM, Créteil, France

-Stryker, Mahwah, NJ, USA

-Unspecified implant brand

-B. Braun,Aesculap, Tuttlingen, Germany, Groupe Lépine, Genay, France

-Groupe Lépine, Genay, France

-Gruppo Bioimpianti, Peschiera Borromeo, MI, Italy

-Zimmer Biomet, Warsaw, Indiana, Serf, Dècines-Charpieu, France

-ATF, Marignier, France, SERF, Décines-Charpieu, France

-Unspecified implant brands (likely multiple)

-Mathys Ltd, Zimmer Biomet,Lima Corporate, Smith&Nephew, Serf, Adler Ortho, Stryker Corporation

-Unspecified implant brands

-Lima Corporate, Villanova San Daniele del Friuli, Italy, SERF, Dècines, France

-Zimmer Biomet, Orthomed E

-Unspecified implant brands
